# Service robots for affective labor: a sociology of labor perspective

**DOI:** 10.1007/s00146-021-01208-x

**Published:** 2021-04-28

**Authors:** Anna Dobrosovestnova, Glenda Hannibal, Tim Reinboth

**Affiliations:** 1 Institute of Visual Computing and Human-Centered Technology, TU Wien, Argentinierstraße 8, 1040, Vienna, Austria; 2 OFAI, The Austrian Research Institute for Artificial Intelligence, Vienna, Austria

**Keywords:** Affective labor, Human–robot interaction, Service economy

## Abstract

Profit-oriented service sectors such as tourism, hospitality, and entertainment are increasingly looking at how professional service robots can be integrated into the workplace to perform socio-cognitive tasks that were previously reserved for humans. This is a work in which social and labor sciences recognize the principle role of emotions. However, the models and narratives of emotions that drive research, design, and deployment of service robots in human–robot interaction differ considerably from how emotions are framed in the sociology of labor and feminist studies of service work. In this paper, we explore these tensions through the concepts of affective and emotional labor, and outline key insights these concepts offer for the design and evaluation of professional service robots. Taken together, an emphasis on interactionist approaches to emotions and on the demands of affective labor, leads us to argue that service employees are under-represented in existing studies in human–robot interaction. To address this, we outline how participatory design and value-sensitive design approaches can be applied as complimentary methodological frameworks that include service employees as vital stakeholders.

## Introduction

There is a gap between social sciences’ discourse concerning emotions at work and how emotions have been addressed by social robotics and human–robot interaction (HRI) communities. Faced with this gap, the more revealing was Monday, 16 March 2020, when the first author boarded a flight to Vienna from deserted Stuttgart airport, one day before Austrian authorities officially closed the nation’s borders in response to fears about the spread of the COVID-19 virus. The flight hosted 6 passengers, excluding the cabin crew. The stark contrast between the warmth of the sunny day and the plane crew’s striving to perform service as usual on the one hand, and the reality of millions of people isolated in their homes, markets plummeting and echoes of personal tragedies across social media on the other, emphasized by the eeriness of an empty plane on an otherwise commonly busy route, was ultimately impossible to ignore. Quick, intense, and straightforward gazes; sincere, but a bit strained smiles exchanged between the passengers and the flight attendants, and other subtle bodily signs—all indicated the shared awareness of how out-of-ordinary the situation was. Once onboard, and with the protocol safety demonstrations performed while locking eyes with every passenger in her sight, one of the flight attendants engaged in a casual, and even cheerful, exchange with a young man sitting a couple of rows in front of the first author.

These seamless transitions between prescribed professional behaviors and improvised engagements—which in other circumstances might be perceived as inappropriate, but now were welcomed as reassuring—served as a perfect illustration of the complexity involved in performing service work; they also indicate the intricate balance between the recognition of one’s own and others’ emotions. Notably, the simultaneous effort to regulate both their own and their customer’s emotions is part of many service employees’ workplace routine. In fact, emotional exchanges, such as described above, are now considered the foundation of the service sector of developed economies (Hochschild 2012). Indeed, following the groundbreaking work of Hochschild (2012)’The Managed Heart’ in the sociology of work, flight attendants are commonly referred to as an example in discussions of the roles of emotions in service labor. In this paper, we draw on this line of research to provide a theoretical account of dimensions of affective and emotional labor in service work. We then relate this account to existing frameworks in HRI that already stress the necessity for more human-centered thinking. Based on this, we offer suggestions for how HRI could respond to the issues and opportunities posed by the centrality of emotions in service labor.

For, service labor requires the ability to display appropriate emotions on demand (Rafaeli and Sutton 1987), to regulate undesired emotions, and to induce desired emotions in others (Penz and Sauer 2019). Such demands are not new compared to industrial labor, but their centrality is (Hardt 1999). This centrality suggests an increased demand on workers; in order to remain desirable job candidates, not only workers’ (manual) skills and expertise are explicitly called for, but their entire personhood—feelings, motivation, enthusiasm, dedication (Negri and Hardt 2005). Emotions are thus treated like resources: they can be deployed and capitalized, much like the feminised service sector workforce (Grossman 2019).

Two concepts are especially pertinent when considering individual and structural implications of this capitalization of emotions in the service sector: namely, affective labor and emotional labor. In sociological and socio-political studies of service labor, the term affective labor is used to analyse socio-economic structures that arise with the passage from modern industrial economies toward postmodern economies (see e.g. Oksala 2016; Prada 2010; Hardt 1999). Within feminist traditions, in particular, affective labor has also been understood as fundamental both to contemporary models of exploitation and to the possibility of subversion (Weeks 2007).

Whereas emotional labor, proposed by Hochschild, is a more specific concept that refers to the work of managing one’s emotions to meet the requirements of a paid work. One reason to practice this management of one’s own emotions is to reliably produce/modify the emotions of others. In this sense, someone’s emotional labor is one aspect of their general affective labor. Both emotional and affective labor stress the increasing demand of a service economy on employees emotional resources.

This paper draws on these lines of research in response to another trend in service work, namely the robotization of socio-cognitive service tasks (Van Wynsberghe 2016; Ivanov et al. 2017). More narrowly, this paper is motivated by the question how professional service robots can be understood in relation to particular aspects of affective labor.

In line with developments in AI, robotics and cognitive sciences, more and more of the so-called “professional service robots” (Mettler et al. 2017) are currently being designed to take on work tasks that were previously reserved for humans (Savela et al. 2017). Professional service robots are a particular kind of robot that is deployed for commercial use in the service sector (Van Wynsberghe 2016), rather than in people’s homes. Wirtz et al. (2018) further differentiate between different aspects of service robots, of which we focus on (1) the physically-embodied robots that, (2) perform socio-cognitive tasks. To be clear, this paper discusses issues surrounding the use of professional service robots but is agnostic about the extent to which this technology will be adopted. That extent will be the result of a complex interaction between how actors across society respond to professional service robots, and how these robots are developed in the face of changes in how we understand their place and role in society (Jasanoff 2004).

Now, given that socio-cognitive tasks (e.g. greeting customers, providing information, sustaining a dialogue) necessarily involve some form of social and affective behavior, the professional service robots we discuss in this paper also belong to the category of social robots. Within human–robot interaction (HRI) these are commonly understood as robotic systems that have the capacity to initiate and engage in interactions with humans. This capacity is emphasized by anthropomorphic design cues and a repertoire of social and affective behaviors (Fong et al. 2003; Breazeal 2003a, b). Further, these features may make social service robots particularly disruptive, because “unlike interactions with self-service technologies, customers perceive that they are interacting with another social entity that is providing services” (Belanche et al. 2020, p. 206).

Notably, key profit-oriented service sectors, wherein attempts at robotization are already taking place, remain caught in the imaginary that certain labors are womens’ terrain (Gutierrez-Rodriguez 2014). These sectors include hospitality and tourism (Ivanov and Webster 2019; Nakanishi et al. 2020), retail (Kamei et al. 2010), healthcare (Metteler et al. 2017; Broekens et al. 2009), and education (Belpaeme et al. 2015). Salient examples of professional social service robots already deployed in profit-oriented service sectors are the robot receptionists in the robot-staffed Henn-na hotel in Japan, the MARIO robot greeting guests in Ghent, the PEPPER robot operating as a receptionist in Hamazushi restaurants in Japan, the BOTLR robot butler in the Starwood’s Aloft, the NAO robot answering questions at Tokyo airport, and the CONNIE robot deployed as a concierge at Hilton hotels (Ivanov et al. 2017; Yang et al. 2020; Gardecki et al. 2018).

In every case, an important distinction between social service robots and human employees remains—despite (gendered) anthropomorphic cues, the apparent fact that robots function as a social counterpart, and their ability to support many types of mundane dialog-based service encounters—professional service robots can only mimic a limited set of emotions and engage in very narrow scenarios of interactions. By contrast, human employees can and do engage in deep acting of emotions (Ashforth and Tomiuk 2000). They are also able to spontaneously step out of the organizationally and culturally prescribed norms about displaying emotions and attending to the emotions of others. In today’s human–robot configurations, this gap is (usually) as obvious, and ways to address it remain elusive.

This paper explores this difference, To do so, we (1) discuss the limitations of the current approaches to emotion for professional service robots; (2) introduce the sociology of labor perspective on emotions in service industries to HRI community; (3) provide some suggestions about how insights from sociology of labor could be integrated into HRI; and (4) summarize why value-sensitive and participatory design offer promising approaches to professional social service robots. We emphasize that human service employees are a group of stakeholders that have been overlooked in HRI, but whom we view as having a central role in the future of professional service robots.

Towards these ends, this paper is grounded in the core assumption that when professional service robots are introduced “in the wild” (Sabanovic et al. 2006), they will inevitably influence how human employees perform emotional labor and how they relate to their work tasks and workplace identity (see e.g. Dobrosovestnova and Hannibal (2021); Tuomi et al. 2020). Insights from the sociology of labor are especially relevant here because they establish a connection with positive and negative implications of service labor, characterized by its reliance on socio-emotional processes for human employees, on individual and on structural levels. To engage with these, we affirm a process-oriented approach (Seibt et al. 2018) to how service robots reshape emotional processes in work environments, and to how people, especially employees, relate to professional service robots.

Throughout, we recognize the economic (see Ivanov and Webster 2019), academic (see Share and Pender 2018), ideological (see Brooks 2013) and pragmatic (see Drexler and Lapre 2019) momenta behind physically embodied service robots. At the same time, we insist that critical perspectives on this trend have pointed out important issues, including several related to the gender norms and identities that such robots materialize (see Schiebinger et al. 2011–2018; Sengers 2018).

We also recognize that affective labor is closely related to discussions of how gender is represented in the design of social service robots. At several points in the paper, we discuss gendered service robots in relation to female service employees, who are particularly associated with emotional labor precisely because they are women (Weeks 2007). To the specific point of robot design, we discuss how various service robots currently deployed in the field (e.g. Pepper, Connie and others) take cues from cultural imaginaries that associate service work with a gendered female form (Carpenter et al. 2009). Problematically, the argument goes, these artifacts reinscribe deprecatory gender norms (Schiebinger et al. 2011–2018). We contend, the sociology of work offers insights about affect generally, and its relation to gender in particular, that offer a basis for an empowering approach to professional service robots.

That being said, our aim with this paper is to stress that affective labor is something that everyone in service work is demanded to master, and which concerns all service employees, as a result.

## Approaches to emotions in HRI

Integrating broader critical perspectives on service labor into HRI requires that we understand the relation between these ideas and current research in HRI. Specifically, we are interested in a set of tensions that arise when professional service robots, designed in line with a particular understanding of emotion, are introduced into naturalistic environments to perform tasks that, for human employees, rely on the complex processes of regulating and displaying of emotion and inducing of emotional experiences in others.

In this segment, we will focus on outlining the first of these tensions: namely, how emotions are approached in HRI; the limitations of these approaches; and how a more process-oriented, interactionist approach is better suited to capture emotions in HRI.

Commonly, approaches to emotion in HRI draw on the basic principles and methods that surfaced in the late 1990’s and early 2000’s (e.g. Brooks et al. 1998), as insights from the social sciences were used to explore and develop more intuitive interactions between robotic systems and human beings. To facilitate and regulate human–robot interactions, the focus was placed on the structure of interactions and on displays of emotions in communication (Reeves and Nass 1996).

In line with these developments, social robots (Fong et al. 2003) are designed to initiate and maintain social interaction in a way that is intuitive for people (Breazeal 2003a, b). That is, the ability to display and recognize emotions as part of non-verbal communication is a central component of the design of such robots. Given the emphasis on displays, current research on the social cues for emotions within social robotics tends to focus on factors such as e.g. facial expressions, gaze, body gestures and posture, bodily contact, and proximity, joint attention, and action.

One consequence of this emphasis on display and recognition of emotions is that social robots have remained predominantly technologically-determined. Vincent et al. (2015) summarise that “[…] robotic design and technological exploration have dominated both the design and the studies on robots”. As a result, constraints on what is technologically possible have meant that professional social robots currently remain designed to complete narrowly defined tasks, and perform roles that are predefined and scripted for a limited range of interactions, typical in the domain of application (see e.g. Campa 2016).

The particular models of emotions that most professional service robots have integrated are similarly limited. Specifically, pragmatic concerns have led most within HRI to adopt computational models of emotion (see e.g. Picard 2003; Rodrıguez and Ramos 2014; Paiva et al. 2015) since these are better suited to being programmed into robots. One common feature of these models is that they are internalist. That is, they understand emotions as internal mental states that are not directly observable. For emotions to become meaningful and useful for social communication, they need to be encoded and decoded based on predefined and accustomed features (Sengers 2018). By extension, internalist efforts tend to model emotional interactions in terms of causes and effects, under the assumptions of straightforward mappings between input and output of emotional information processing in both robots and humans (Boehner et al. 2005). As a result, the idea of inducing emotions “in'' another agent has dominated work on emotions in relation to service robots within the internalist framework until recently (Fong et al. 2003; Leite et al. 2013).

A closer look on the successes and failures of such work in HRI reveals than an internalist understanding of emotions makes it difficult to study situated behaviors and interactions with robots (Boehner et al. 2005). In part, this is because emotions, and affect generally, are difficult to study in terms of internal component processes (Alac 2016). A more fundamental limitation to internalist approaches, though, is that emotions have increasingly come to be understood as complex, dynamic, and situated within the cognitive sciences. This has the consequence that the responsibility for appropriate emotion expression and interpretation is now seen to be shared between humans and robots.

In the context of social robots, Jung (2017) further challenges what he calls the “signaling approach” on the grounds that in order to understand emotions and their regulation in human–robot interactions, attention should lie on the joint activities between participating parties. This leads to a perspective on emotions that sees them as an effort of coordinating affect in social interactions. In this framework, emotions are never simply expressed and (mis)understood, but always interpreted.

Importantly, this view emphasizes the ways emotions are shaped by the structural and political dimensions that are implicit to interaction. For us, this means accepting the insufficiency of a reductionist view wherein every single expression or interpretation of emotion can be accounted for in computational terms since the complexity of any interaction understood in the terms described above makes it computationally intractable.^1^

Along these lines, numerous authors argue that approaches to emotions within social robotics should likewise focus on the dynamics of the interactions themselves, rather than on internalist models of emotion (e.g. Boiger and Mesquita 2012; Boehner et al. 2005; Höök et al. 2008). From this perspective, humans take an essential role by the continuous, and active interpretation of situated emotions.

However, humans tend to be understood as being more passive than this in much of the contemporary HRI. As far as emotions are concerned, the dominant, internalist understanding of emotions as discrete, internal and objective states leads to an emphasis in HRI on questions like whether a certain emotional display was correctly decoded by human participants in interaction (see e.g. McColl and Nejat 2014).

This explains why the focus in the development and evaluation of professional service robots is still mostly on whether people find the robot technically challenging to operate in a naturalistic environment (see Pinillos et al. 2016), or on measurements of trust (see van Pinxteren et al. 2019), engagement and comfort (see e.g., Rodriguez et al. 2015; Tung and Au 2018), perceived security and intelligence of the robot (see e.g., Tussyadiah and Park 2018); perceived role of the robot (see e.g. Ljungblad et al. 2012); and willingness to accept it (Savela et al. 2017; Vishwanath et al. 2019). Concerning the kinds of interactions being imagined here, this means that the human aspect of HRI is still predominantly understood through the prism of robots and how they are perceived by people.

In response, an interactionist perspective calls for questions that also consider how people perceive themselves in relation to the robot since the perceived relationship shapes how humans make sense of the robot and guides the narratives constructed about it (Goodrich et al. 2018). Further, the interactionist perspective invites questions about how the introduction of a robot into a naturalistic environment, where tasks and space are shared with people, reshapes the social and emotional processes between people.

When it comes to emotion research, this perspective also raises the issue of the specific context in which an affective and social interaction takes place and that of the social demands that allow for certain emotions to be expressed as an appropriate response in a given situation (Fischer et al. 2019).

In line with this, we argue that the sociality of robots is not determined by, and limited to, a technological innovation that can be added to increase the robot’s appeal and how intuitively easy it is to interact with the robot (Breazeal 2003a, b). Instead, it relies on the fundamental aspects of the robot being present in a social situation, and on its perceived role in society (Dautenhahn 2007; Boehner et al. 2007). This perspective suggests overdue on moving away from assessing social robots in terms of their technological capabilities, as well as from the efforts directed at getting individuals and society at large to accept these capabilities for what they are. We also call for increased awareness with respect to attempts to deploy technology to fix fundamentally social and political problems (cf. Morozov 2013). To do so, it will be crucial to explore how technology is shaped by social and cultural factors, and, in turn, how these are re-shaped in the use of technology by people (Feenberg 2010).

As with the introduction of any technology, it is not at all apparent what effects professional social service robots might have (Jasanoff 2004). Given this paper’s emphasis on the work of service sector employees, it goes on to consider particular questions such as: how people change the ideas they have about their workplace identity; how they experience their emotions with respect to externally prescribed and programmed into a robot; last but not least, how the very structure of social relations and emotions at work is affected with the introduction of a professional service robot. In our view, to answer these questions, we must first understand the demands with respect to emotions that are placed on human employees in service sectors, as well as some positive and negative implications these may have.

## Sociology of labor perspectives on emotions

The challenges to current approaches to designing and studying emotions for professional service robots are related to the need for HRI to better understand the emotional processes that human employees in service sectors are engaged in, as well as the relational structures that arise from these. In this segment, we will outline some of the key insights about emotion-related processes in service labor from the sociology of labor perspective. We will also discuss how, in our view, professional service robots might disrupt and redefine these processes.

In sociology and gender studies of labor, emotion production, regulation and management is recognized as central to service work (Rafaeli and Sutton 1987; Negri and Hardt 2005; Hardt 1999; Swenson 2011; Penz and Sauer 2019). These processes are commonly referred to under the umbrella term of “affective labor”. While affective labor is also present in the background of industrial labor (Sengers 2018), it is especially critical for the service sectors that define post-Fordist economies, given their heavy reliance on the production of immaterial goods (Weeks 2007). Following Penz and Sauer (2019), in these economies, “not only do the social standards, the rules, and the norms of society change but also the people subjected to the social order, the subjectivities undergo changes when emotional habits, the affective resources or’affective capital’ of people, are at stake and targeted in new ways” [p.2]. In relation to this, we understand affective labor as a subcategory of immaterial labor referring to waged work that relies on the production and modification of emotional experiences. In that, affective labor is rooted in the foundation of human contact and interaction; its “products” are relationships and emotional responses (Oksala 2016).

Within this broad scope, we find it useful to highlight what Hochschild (2012) defined as “emotional labor”. Note that this distinguishes emotional labor from affective labor. This distinction is important because emotional labor addresses processes that pertain to affective labor on the individual level, in contrast to the collective and structural levels. We follow Hochschild (ibid.) in arguing that emotional labor occurs when an individual produces or modifies their emotion experience and emotion display in order to meet workplace demands that include, among others, production and modification of emotional experiences in other people.

With respect to Hochschild’s work, there are two central aspects we wish to highlight, namely that (1) when we emote, we do not always express whatever is congruent with our internal state at a given point in time, but we “curate” (i.e., control and present our emotions) in accordance with the societal expectations and norms, and that, (2) the norms and values operate on our emotions via feeling rules, understood as the standards that are used to determine what is “due” in each relation and each role. Hochschild defines this process of curating one's emotions via feeling rules as *emotion work*. Emotion work becomes emotional labor when it is performed to meet workplace demands, and in line with externally prescribed organizational norms and established practices of work performance.

Notably, Hochschild has been extensively criticized, among others, for taking on an essentialist position i.e., for assuming that there is some “true” or authentic emotional self (Weeks 2007). In some ways, this is reminiscent of the internalist understanding of emotions in HRI, which also assumes the existence of a discrete, but also objectively-knowable emotion. Despite this similarity and critique, Hochschild’s work has proven itself instrumental for the fields of sociology and work psychology. In this paper, we refer to the concept of emotional labor specifically to highlight the difference between how service employees feel and how they are expected to appear to feel. The conceptualization of this difference in terms of the two strategies surface and deep acting (Hulsheger and Schewe 2011; Ashforth and Humphrey 1993)—is another contribution that stems from Hochschild’s work and that we will draw in our follow-up discussion. Deep acting refers to the situations of experience and display of emotions wherein the professionally required and the authentic feelings of an individual align. By contrast, surface acting is oriented toward behavioral response, and can be described as an effortful process of managing one’s emotions (Hulsheger and Schewe 2011).

Important for the present discussion, deep acting and surface acting of emotions as part of service work are associated with the so-called emotion-rule dissonance (Hulsheger and Schewe 2011). Emotion-rule dissonance arises in the situations of conflict between an inner state of an individual and the externally prescribed feeling rules. In other words, deep and surface acting represents two strategies of emotional labor that workers can engage in as a response to the perceived need to regulate one’s emotions to meet the demands of the workplace situation.

### Social service robots and affective labor

This section is concerned with how professional service robots may contribute and disrupt individual emotional labor, and the collective socio-affective structures in service sector workplaces. In this regard, we highlight three points: (1) the relationship between the narrow repertoire of social and affective behavior of service robots and the potential burden on emotional labor of human employees that may arise from such limitations; (2) the contribution of service robots to established corporate “feeling rules” and (gendered) stereotypes about service work; (3) the re-shaping of social and affective structures between people as an outcome of interactions with the robot.

Above, we discussed how decisions about social and affective behaviors in professional service robots deployed in profit-oriented service sectors are shaped by the technological constraints on the robot's design and by internalist models of emotions in HRI. To these, add dominant codes and narratives about service work, as well as the general goals (i.e., increased profitability; standardization of service) that the current deciding stakeholders pursue with the robotization of service tasks (the Henn-na hotel is one example—Osawa et al, 2017). Taken together, these factors result in robot designs that represent a significant reduction in complexity (Kaerlin 2015) of social and affective behaviors, when compared to the complexity of behaviors in human employees performing similar tasks.

The reduction in complexity suggests a tension between human employees, whose emotions is guided—but not determined—by organizational and cultural norms, and the fixed and narrow behavior repertoires of professional service robots; and it raises a concern about how complexity reduction might contribute to the reinforcement of stereotypes and dominant representations of affective labor and of service workers. And how such reinforcement, in turn, may place additional burden on service employees’ emotional labor (see e.g., Stock and Merkle 2018).

For example, gender is purposefully ascribed to some social robots (see e.g., Powers et al. 2005; Stroessner and Benitez 2019) as a way of ‘simplifying’ interactions, since, it is hoped, gendered codes will steer users towards certain types of interpretations (Coeckelbergh 2019; Moran 2019). This is especially the case for interactions that are assumed to be associated with the robot’s assigned “gender” (Shaw-Garlock 2017). Problematically, this affects more than just a reduction in complexity. Namely, gendering professional service robots perpetuates normative ideas about “women’s work” (ibid.), and about the demands upon their affective labor that further burden female employees (Weeks 2007).

Apart from an increased burden on emotional labor, another concern is related to the processes of change in organizational norms and practices of performing emotional labor that may be halted or significantly reshaped with the introduction of a professional service robot. Precisely, Ashforth and Humphrey (1993) argue that service employees are able to step out of the roles prescribed by organizational culture to communicate the nature and depth of their personal convictions; or to respond to a non-trivial situation. If these instances of stepping out accumulate, they may lead to reshaping of organizational identities and to formation of new feeling and display rules. The openness to change and resistance (Tsianos, and Papadopoulos 2006) on the one hand, and the narrow and fixed behaviors of professional service robots on the other hand, introduces another tension that requires further (empirical) investigations.

That being said, it is important to remember that emotional labor is not always associated with increased negative outcomes such as emotion-rule dissonance and, as a result, increased stress, burnout and workplace dissatisfaction. To the contrary, in many instances, service workers engage in deep acting voluntarily and take pride in the emotional labor they perform (see e.g. Penz and Sauer 2019). For the scenarios where a professional service robot is introduced, this invites questions how to: (1) avoid placing additional burden on human employees’ emotional labor, (2) enable scenarios and interactions where human employees would engage in deep acting voluntarily.

The possibility of the latter is suggested in the study by Tuomi et al. (2020) where, in the role of differentiators, front-line restaurant employees spent more time on the floor interacting with customers. According to the study’s authors, the increased time to interact with customers could lead to positive outcomes on individual and organizational levels.

However, it did not prove to be the case in the Henn-na hotel, which failed with regard to allowing its employees to focus on customers in a similar way, because robotization added routine maintenance tasks and the managing of guests' interactions with the robots to the employees' workload. This outcome is not only the opposite of the Henn-na managers’ goal, but also runs contrary to the post-work imaginary’s shift towards work-as self-actualization, as associated with robotization (Hester and Srnicek 2019). To this point, Wajcman (1991) work may be instructive, because it illustrates how norms and standards can be shifted following the introduction of a technology, in a way that places higher, not fewer, demands on the people it was designed to benefit.

Another important insight stemming from the sociology of labor perspective on emotions in service work concerns spontaneous positive externalities, such as networks, attachments, passions. For instance, by extending Foucault’s concept of biopower, Hardt (1999) argues that affective labor is not only an instrument of subjectivation and dominance, but is a possibility for solidarity because it enriches production to the level of complexity of human interaction.

To this point, previous work hints at ways in which robots may reshape social dynamics at the workplace. A curious example is a snack delivering mobile robot that mistakenly called a participant by another participant’s name. In this situation, a narrative had already emerged in the office in which the robot was deployed, that the robot preferred the participant whose name it called, though the logs show the robot spoke no more to this participant than to anyone else. The robot’s mistake was variously interpreted by the participants as “flirting with” or having a crush on the participant whose name it called out (Severinson-eklundh et al. 2003). Reminiscent of Höök et al. (2003) affective technologies and other devices discussed in Boehner et al. (2005), the robot reshaped social dynamics by opening up a space for employees to come together and exchange feelings and impressions.

Another example to consider in relation to being affected by and affecting others is Castaneda (2001) analysis of robot Cog’s skin which serves as a boundary to protect the device from contact and harm. Interestingly, a parallel could be drawn between touch, in Cog case, and sexual harassment in the service sector. As is the case with Cog, touch reflects a boundary, and serves as a paradigmatic example of a situation in which the particular environment frames actions in particular ways (Good and Cooper 2016). By drawing this parallel, we recast Cog in relation to sexual harassment in a way that raises questions about how professional service robots could act to intervene upon sentiments like it being service employees’ job to be “friendly” (ibid.), even when they feel threatened (as Cog is by any touch at all).

Significantly, while affective and emotional labor relate to all service employees, not only women, this example illustrates they do not do so in the same way (Oksala 2016). Notably, it is these moments of difference that lead Suchman (2007) to point out that the design of robots could be a site of resistance, at which normative expectations are intentionally confounded in order to interrupt the reiteration of a limited and essentialized femininity. For example, making Cog’s vulnerability visible, and that of its successors deployed in the service sector, might be part of an intervention that problematizes casual, unwanted sexual advances against service workers (cf. Knox 2019).

To summarize, fundamentally, what affective and emotional labor offer is a set of insights, questions and methods to understand the affective dimension of the workplace. These, we argue, are invaluable to HRI studies because they raise some of the fundamental challenges and questions that will have to be addressed for any professional service robot to be successful. In the next section, we will discuss methodologies that might allow HRI to respond to these challenges.

## Methodological implications for HRI

Concerning all social robots, Seibt et al. (2018) crucial insight is that research, design, and development do not produce robots, but interactions. The shift in focus from robots as stand-alone artifacts to interactions makes for the central question: how can desired interactions be understood and designed? As far as service labor is concerned, this requires including knowledge and methods produced especially to study this socio-cultural domain (ibid.).

The previous section summarized valuable theoretical and empirical insights from the sociology of work, and how these, in our view, relate to professional service robots. In this section we will rely on these insights for a more methodology-oriented discussion about how design and evaluation of professional service robots can be improved by integrating these perspectives.

We point to three immediate practical concerns, namely that (1) we must recognize service employees as central stakeholders in relation to professional service robots, (2) recognizing service employees as stakeholders entails a shift in the primary methods used to study HRI in the context of professional service robots, and (3) this opens up service robots as sites for intervention into a variety of socio-political issues, in particular concerning (workplace) identity and gender. Practically, such interventions leave considerable tasks on designers’ tables (Baker 2018), who, we will suggest, could apply particular design approaches that offer a break with the current, more technologically-oriented methods.

Part of the latter task, as we have also tried here, is reflecting on how current design methods reproduce dominant sociotechnical norms, and how we respond to this. Consider that the relative absence of service employees from studies about professional service robots illustrates that the design of post-industrial society is not determined, but contingent (Feenberg 2010). This suggests that dominant design principles will tend to focus on (and then reify) the values and interests of the hegemonic cultural imaginary (in relation to robots, see e.g., Kovacic 2018). In the concrete case of professional service robots this would (and does) establish factors like narrowly-defined efficiency, and other performance-related outcomes as the central measures of success (see also Sabanovic 2010). This has come at the expense of considering demands on workers, as we have illustrated in reference to affective and emotional labor.

One suggestion to address this challenge is by democratizing technology by inviting more actors to participate in the design process. For “technology has beneficial potentialities that are suppressed under capitalism and state socialism. These potentialities could be realized along a different developmental path where power is more equally distributed” (Feenberg 2010, 71 cited in Coeckelbergh 2020, 91). One way to distribute power more equally, when it comes to professional service robots, is by permitting service employees a louder voice in their design and in decisions about their deployment.

Within HRI, authors including Severinson-Eklundh et al. (2003) previously raised similar concerns, albeit from a different angle, about the degree to which employees are involved in the decisions and processes leading up to the potential deployment of service robots at their workplace. While early inclusion of human co-workers in this process has also been identified as a crucial element of attitudes to robot co-workers (Kaufmann et al. 2019).

In cases when robots are already deployed though, we point to Usability, Social Acceptance, User Experience, and Societal Impact (USUS) evaluation framework as the most extensive’tool’/evaluation method in HRI, also because it does take into account more sociological perspectives (Weiss et al. 2009; Weiss 2010). However, the framework’s focus, at first, was primarily on the investigations of representations and expectations concerning “sociotechnical imaginaries” (Jasanoff 2004), as informed by narratives generated in literature, mass media, as well as by the visions of the future by researchers and developers. As a result, studies continued to give little attention to the lived experiences of people interacting and co-working with humanoid robots long-term. While an additional recent publication (Wallström and Lindblom 2020) was intended to “extend/improve” USUS, we do not agree this was accomplished, since the extension reverted to a more engineering/design-based view.

Given these limitations, the current formulation of USUS appears insufficient. However, there is a place within USUS to engage with the issues around affective and emotional labor. Specifically, these could be addressed within the evaluation factor “Societal Impact”, which Weiss et al. (2001) define as: “[…] all effects the introduction of robotic agents causes for the social life of a specific community (taking cultural differences into account) in terms of quality of life, working conditions and employment, and education”. Thus, USUS offers a way to integrate the points raised in this paper with other factors in terms of which professional service robots might be evaluated.

What USUS does not offer is a set of methods with which to integrate the points we have raised in the preceding segments into the design process of professional service robots. However, the fact that we urge HRI to place greater emphasis on employees as stakeholders in itself suggests a way to approach this challenge. For, one motivation for this shift in emphasis is the insight that employees bring a particular set of values to their workplace, which are key to understanding how they relate to and interact with professional service robots, as well as with other people. Thus, values re-emerge as a prism through which to investigate the relations between such robots and service employees, much like values are an important aspect of work on affective and emotional labor, for example in relation to stepping out of corporate feeling rules (Ashforth and Humphrey 1993).

Now, “observing values within a system is a complex endeavor whereby the promotion of one value may be fulfilled while at the same time there is a tradeoff with another value.” (van Wynsberghe 2013). While this conflict cannot be resolved within HRI, what we can do is develop methods for mapping and reconciling different values. In fact, this is the core of value sensitive design, which offers a range of specific methods through which insights from the sociology of work can be incorporated.

Specifically, “Value Sensitive Design is a theoretically grounded approach to the design of technology that accounts for human values in a principled and comprehensive manner throughout the design process” (Friedman et al. 2013). Within the domain of HCI, for example, Friedman et al. (2017) identify 14 value sensitive design methods, including one’s related to particular stakeholders, values and the co-evolution of technology and society.

In relation to HRI, Aimee van Wynsberghe (2013, 2016) has been working on the value-sensitive design approach as a means for creating a framework tailored to care contexts. For values sensitive design, it is crucial that, “if ethics is to be included in the design process of robots, one must first identify the moral precepts of significance followed by an account as to how to operationalize said precepts” (van Wynsberghe 2013, p.408). This raises questions about what are the specific issues pertaining to corporate environments. In our view, part of the challenge is that these questions extend beyond HRI—beyond any single discipline/perspective in fact—meaning they require collaborative effort and interdisciplinary work.

A more fundamental challenge is that value sensitive design in general, and van Wynsberghe (2013, 2016) as a distinguished example of it, assumes the existence of a particular set of identifiable values. Whereas, research on professional service robots does not currently have a literature about corresponding values to draw on. In fact, studies in the sociology of work even emphasize local aspects like corporate feelings rules as a crucial aspect of affective and emotional labor. This suggests that a value sensitive design will need to be complemented by another method well-suited to bringing out values and to empowering service employees to voice their own perspective on them.

Here, a participatory design stance may offer a solution. This approach has already been well developed and used in HRI (see e.g. Bertel et al. 2013; Björling and Rose 2019), and using PD would enable us to likewise explore, on an empirical ground, open and interpret issues like those discussed above in relation to emotion-rule dissonance. It is promising that, in recent years, participatory design approaches are gaining popularity in the HRI community (Lee et al. 2017).

Usefully, one aspect of PD approaches involves reducing emphasis on making robots more sophisticated technologically. Instead, it offers an opportunity to engage in a conversation about the necessity and desirability of such technological sophistication in the first place. Here, the strength of a PD stance is that it aligns stakeholders’ views on what is useful/needed/desired/valued. In this way, PD breaks with the practice of engineers starting the design process with introspection (Forsythe 1999).

Interestingly, a PD approach also does not foreclose any particular abilities of the robots. For example, while the very necessity of attempts to achieve emotions that comes close to that of humans is contested within the HRI expert community (Sharkey and Sharkey 2010), a PD stance empowers service employees to take a larger role in this conversation. Broadly, in response to the complexity, open-endedness and “interpretative flexibility” (Howcroft et al. 2004 cited in Van Wynsberghe 2013) (for example, see Ljungblad et al. 2012; Vishwanath et al. 2019) this suggests that HRI can leave room to engage in conversation about people’ experiences with robots (Turkle et al. 2006) and about specific issues like human vulnerabilities (Coeckelbergh 2013).

In the context of professional service robots, taking a PD stance reiterates that people, who are to share work spaces and work tasks with service robots, can and should participate in the decision with respect to the role they think the robot ought to take, and whether and how the robot ought to perform affective and social behaviors.

One especially interesting situation might arise if participants in the PD process of a professional service robot appear to decide that their concerns are not to do with technology design/development at all. In this situation, it is our view that a PD allows/should allow participants to reformulate the problem since problem-framing is a central aspect of the methodology in the first place. In fact, this might be an avenue towards the more critical aspect of the approach we outline in this paper. For, leaving the fundamental framing of the problem open, as PD does, allows a service employee, or any other individual, to identify the issues outside a “technology-fix”.

Further to this point, and bearing in mind Wajcman (1991) warnings about the uncertain emancipatory promises of technological artifacts, we contend that the key in relation to professional service robots is designing empowered interactions, above and beyond designing artifacts, that can counteract existing norms. One point to come back to, as a result, is that the openness of the problem framing makes it even more important to have an interdisciplinary approach to the process of developing robots for affective labor since issues here overlap with those in the social sciences and humanities.

Indeed, we consider having shown that the development and application of professional service robots is a fabric of layered and overlapping issues across disciplines. Inspired by Seibt et al (2018) we hope that by integrating these approaches in this paper, we have contributed to transforming some central notions about effect, emotions and service labor in HRI.

## Conclusion

To summarize, this paper makes a number of contributions to HRI research into professional service robots.

First, it suggests how affective perspectives, as informed by sociology of labor, could be further integrated in HRI research. We pointed out that popular models of emotions in social robotics are internalist and rely primarily on mapping the internal states of a robot onto predefined categories of emotion signaling behavior. We highlighted interactional approaches to emotion instead and pointed out how these shift the focus towards interactions, rather than artifacts, and to sociality that cannot be scripted in any straight-forward way. In our view, the crucial aspect of this shift is that it moves HRI away from an understanding of humans as passive users, understood through the prism of robots, to humans understood as active participants in a dynamically unfolding social interaction.

Second, we introduce a social and labor science perspective on human–robot interactions in work settings, which, unlike the previous point, has not received much attention in HRI thus far. We argued that both current studies of professional service robots in HRI leave out more nuanced psychological, relational and political implications of social robots for the human labor force. For, affective and emotional labor raise a number of crucial concerns.

Third, we responded to these omissions by considering how particular challenges could be approached within HRI. One challenged concerned emotion-rule dissonance, which makes apparent the extent of service sector employees emotional labor. An approach within HRI would be to consider whether there are opportunities for professional service robots to facilitate deep acting, or if there are other ways these robots could enable service sector employees to express deep-felt emotions.

In turn, we considered that such displays are guided by and, on occasion, break out of corporate feeling rules. In relation to this, we discussed professional service robots understood as a reduction of complexity. Especially interesting future work in this regard might deal with what would be involved in getting a service robot to act accordingly and in (in)appropriate violation of such rules. Moreover, robots’ presence as social actors within service-oriented workplaces raises questions about how these robots might act to solidify or modify existing feeling rules. That, if nothing else, establishes them as normative technologies that give practical effect and meaning to certain ideas and beliefs that they embody (Jasanoff 2004), for example about what to feel when and how to express it.

We grounded our proposal for the inclusion of these more relational and experience-based aspects of co-working with service robots in participatory design and value-sensitive design approaches. We also discussed the USUS as a potential candidate framework for the evaluation of socio-relational factors for the robots already deployed in the field. We emphasized that these methodologies offer an approach to the introduction of professional service robots that would recognize service employees as central stakeholders, and also empower them as co-designers of their potential robot co-workers.

We hope that our paper contributes in a meaningful way to the strengthening of interdisciplinary work in HRI. In it, we have offered a theoretical account of dimensions of emotional and affective labor in service work and related these to existing frameworks in HRI that already stress the necessity for more human-centered thinking. Throughout, we provided suggestions for how HRI could respond to the issues and opportunities we have raised and invited further discussion about how insights from social sciences can enable more responsible and aware integration of service robots in real-life settings.

A particular concern has been that professional service robots are embedded in broader socio-political issues like identity and that these robots can be a site of intervention if transdisciplinary research succeeds in empowering stakeholders to co-design potential future robotic co-workers.

## Footnotes

1 In fact, some critics of the internalist view insist that the focus on complex computational models is misguided in several important ways, in particular its focus on information, rather than affect (Boehner et al. 2005).
